# Effects of hMASP-2 on the formation of BCG infection-induced granuloma in the lungs of BALB/c mice

**DOI:** 10.1038/s41598-017-02374-z

**Published:** 2017-05-23

**Authors:** Xiaoying Xu, Xiaoling Lu, Xingfang Dong, Yanping Luo, Qian Wang, Xun Liu, Jie Fu, Yuan Zhang, Bingdong Zhu, Xingming Ma

**Affiliations:** 1 Department of Immunology, School of Basic Medical Sciences, Lanzhou University, Lanzhou, 730000 China; 2 Institute of Pathogenic Biology, School of Basic Medical Sciences, Lanzhou University, Lanzhou, 730000 China; 3 Key Lab of Preclinical Study for New Drugs of Gansu Province, Lanzhou, 730000 China; 4 The First Hospital of Lanzhou University, Lanzhou, 730000 China

## Abstract

Tuberculosis, caused by *Mycobacterium tuberculosis*, affects the functions of the lung and causes high morbidity and mortality rates worldwide. MASP-2 is an executioner enzyme, which plays an essential role in the activation of lectin pathway. In our previous studies, the MASP-2 played a dual role in promoting the progress of lesions in BCG-infected rabbit skin models. However, the really effects of MASP-2 on tuberculosis are unknown. The aim of this study was to investigate the effects of MASP-2 in granuloma formation with BCG-infected mice. Compared to the control group, rAd-hMASP-2 treated group showed increasing in survival rate of BCG-infected mice (P = 0.042), and decreasing of bacteria loads (P = 0.005) in the lung tissue. MASP-2 displayed a protective efficacy in BCG-infected mice, which promoted the activation and recruitment of macrophages and lymphocytes to the granuloma. Moreover, the data obtained from the ELISA and RT-PCR demonstrated that mRNA expression for IL-6, CCL12, CCL2 and cytokines of IFN-γ, TNF-α in lung were significantly elevated by treatment of rAd-hMASP-2. Those findings provided an evidence that MASP-2 may be as a newly immunomodulatory in targeting granuloma formation, which displayed a potential protective role in control of tuberculosis.

## Introduction

Tuberculosis (TB) now ranks alongside HIV as a leading cause of death worldwide. In 2014, 9.6 million people are estimated to have fallen ill with this disease (WHO Global Tuberculosis report, 2015). The pathology of TB is thought to be mainly immune mediated rather than a result of direct cytopathic effects of the infection^1, 2^. The formation and maintenance of granuloma are central to the development of the pathology of TB in murine models and human^3–6^. The granulomas begin with the recruitment of monocytes to infection site where they engulf *mycobacteria*. Then the successive macrophages and other immune cells aggregate with the infected cells to form granulomas^7, 8^. The progression of the active TB disease, characterized by an incensement of granulomas in size, number, and distribution, reflects an inability of the host to effectively eliminate bacilli or clear shed antigens^9, 10^. However, some evidences indicate that the granulomas are critical component of the protective cellular immune response, serving a vital role in pathogen containment^11^. The mycobacteria-driven mechanisms that promote granuloma formation are not clear which hamper granuloma as targets for novel therapeutic strategies to control TB.

The complement system is an integral part of innate immunity that bridges the innate and adaptive immune systems^12^. The lectin pathway (LP) is initiated upon target recognition by pattern recognition receptors such as mannan binding lectin (MBL), CL-11 or ficolin to the carbohydrates on the surfaces of viruses, bacteria, and parasites, which in turn leads to the activation of the MBL-associated serine proteases (MASPs)^13^. The three MASPs, termed MASP-1, MASP-2 and MASP-3, are known. Additionally, MASP-2 alone is capable of initiating complement activation by sequential cleavage of complement C4, and thus triggering the lectin pathway (LP)^14^.

Previous studies have demonstrated that many virus and parasite avoided the complement lectin pathway via mimicking complement inhibitors or binding to host complement inhibitors^15–22^. Such as *Trypanosoma cruzi*, which is the causative agent of Chagas disease, the study found that it resisted the complement lectin pathway -mediated killing by expressing surface receptors, which inhibited MASP-2 cleavage of C2 factor and thereby invaded the host cells to progress in infection^23^. Recent studies have shown that some bacteria, such as *Salmonella enterica* and *Porphyromonas gingivalis*, produced proteases to destroy MASP-2 and hence stopt the complement cascade^24^. However, *M. tuberculosis* is highly adapted to living inside the phagosome of macrophages and it may activate complement to promote its uptake into phagocytes. Carroll *et al*., for the first time, demonstrated that M. bovis BCG binding of L-ficolin from human serum leads to MASP-2 activation which would mediate C4b2a formation^9^. The evidence indicated that MASP-2 was activated by BCG *in vitro* and played role in killing *mycobacteria* in lectin pathway. We have previously studied the effects of MASP-2 on liquefaction and ulceration using a rabbit-skin model of BCG. And found that the MASP-2 induced a dual effect on BCG-infected rabbit skin models, which promoted both the formation and maintenance of granulomas and reduced the survival of the *mycobacteria* within them^25^. However, the mechanism of MASP-2 on tuberculosis is unknown. The BCG-infected mouse model has been used extensively in understanding cell immunity and the granuloma formation in tuberculosis^26, 27^. In this study, we injected an adenoviral vector of encoding human MASP-2 (rAd-hMASP-2) into BCG-infected mice and evaluated the survival, examined the bacterial loads and immune response in the lung to assess the potential effect and mechanism on tuberculosis granulomas.

## Results

### hMASP-2 treatment promoted survival in BCG-infected mice

**Figure 1:**
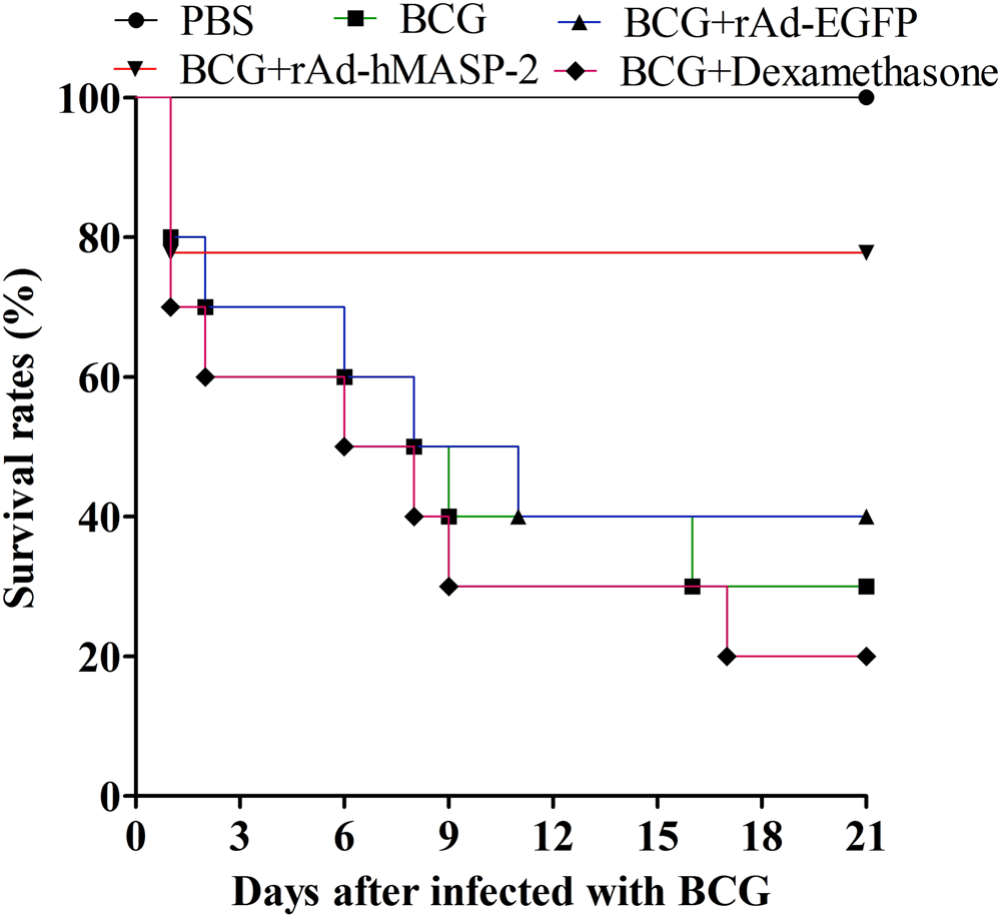
The 21-day survival rate of BCG-infected BALB/c mice with or without rAd-hMASP-2 treatment. BALB/c mice were intratracheally subjected to BCG and they were treated intratracheally with rAd-hMASP-2, rAd-EGFP or dexamethasone, respectively. The survival time and rate were recorded for 21 days in various groups. Statistically significant difference as compared with various groups by Pearson Chi-square test, n = 10 per group, P = 0.037. As compared with the rAd-EGFP group, rAd-hMASP-2 group showed statistically significant difference, n = 10 per group, P = 0.042. Data collected from ten mice per group were expressed as means ± SD.

The survival rate was recorded at 21th days post-infection in various groups. In the BCG + rAd-EGFP group, more than half of animals had died (40%), by which only 20% in the BCG + rAd-hMASP-2 group had died. As compared with the rAd-EGFP group, rAd-hMASP-2 group showed statistically significant higher than control groups in the survival rate (P = 0.042, Fig. 1).

### hMASP-2 treatment reduced the bacterial load in BCG-infected mice

**Figure 2:**
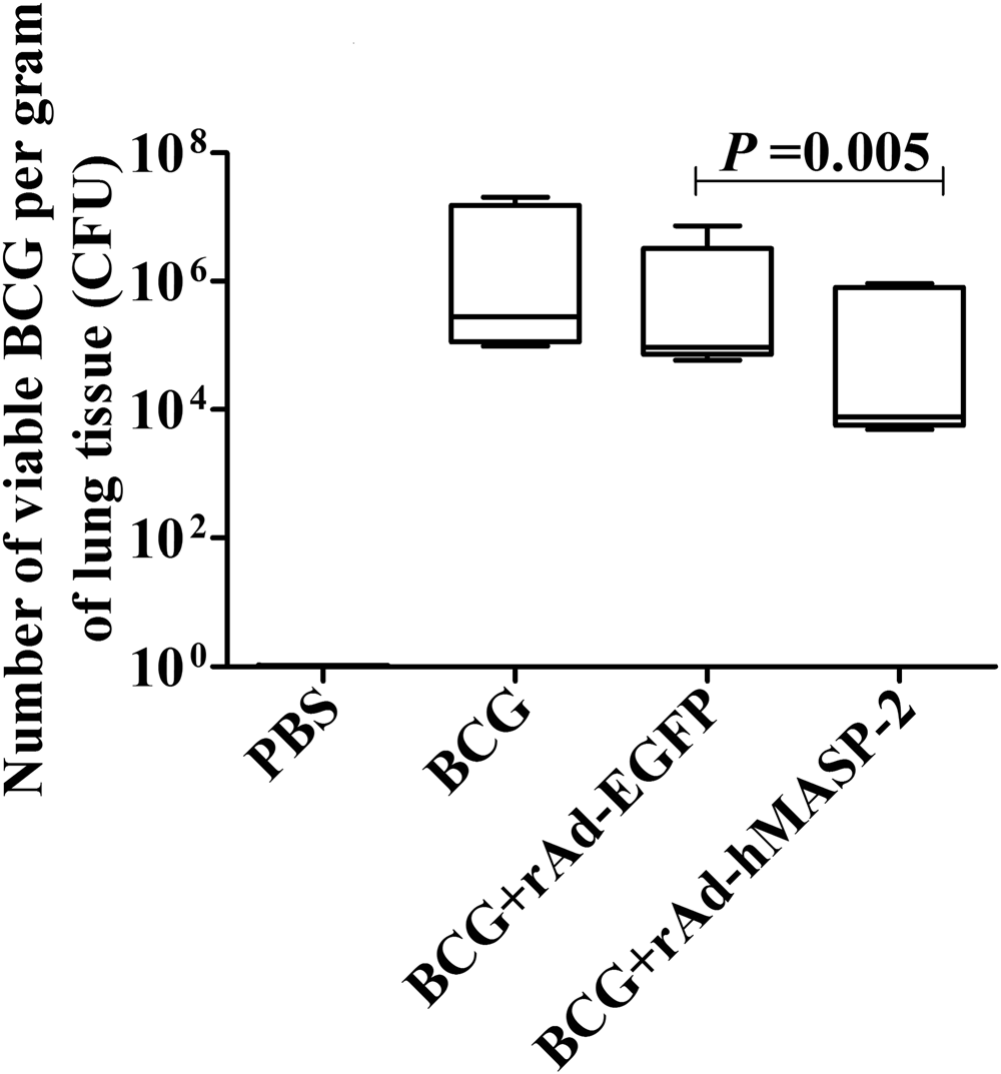
Number of viable BCG per gram of lung tissue. Twenty one days after infection, the number of tubercle bacilli per gram of lung tissue in the mice was determined in each group, n = 5 per group. Data represent mean values of conditions performed in duplicate.

After infecting for 21 days, the bacillary loads were determined. The CFU from the BCG + rAd-hMASP-2 group was about 3 × 10^5^ ± 4.5 × 10^5^ CFU/g, which was ten times less than the rAd-hMASP-2 group (1.8 × 10^6^ ± 3.1 × 10^6^ CFU/g). Similarly, the bacterial loads in BCG + rAd-hMASP-2 group was significantly lower than that in the BCG + rAd-EGFP group (P = 0.005, Fig. 2). There is no statistical differences between BCG(6.4 × 10^6^ ± 9.2 × 10^6^) and BCG + rAd-EGFP(1.8 × 10^6^ ± 3.1 × 10^6^) groups (p = 0.068).

### hMASP-2 treatment enlarged granulomas area in lung tissue

**Figure 3:**
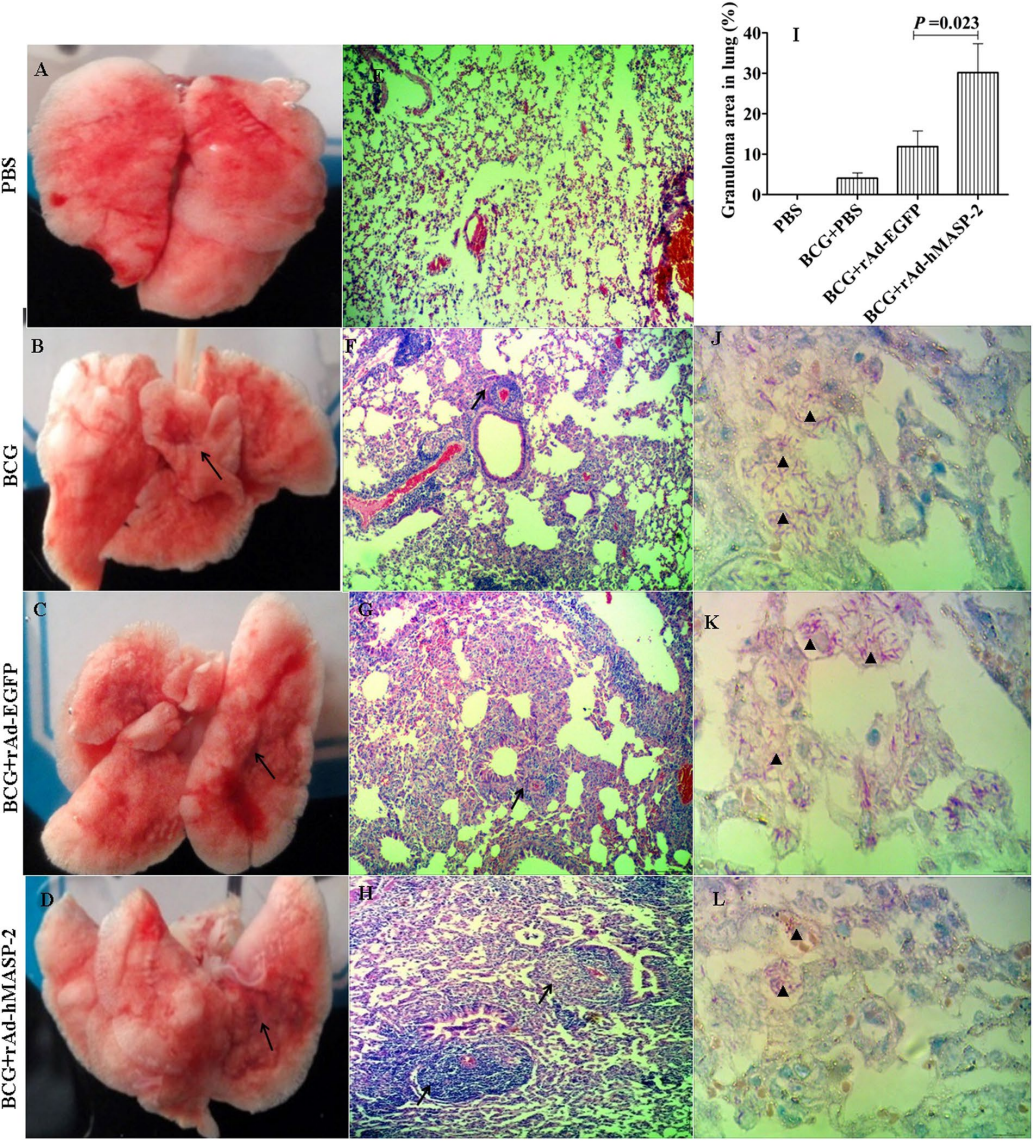
Comparison of the lung lesions in BALB/c mice. (**A**~**D**) Macroscopic images of the lungs at day 21 from 4 representative mice. (**E**~**H**) Histopathological analysis of hematoxylin-and-eosin (HE)-stained lung sections from 4 representative mice. Granulomatous lesions in BCG-infected mice (**B**~**D** and **F**~**E**) in contrast to normal lung tissue in PBS mice (**A** and **E**) at day 21 (arrows: granuloma lesions, ×200). (**J**~**L**) ZN-stained lung recuts showing bacilli (▲) growing (**J**~**L**) in BCG-infected mice and no bacilli within the uninfected mice. (**I**) Percentage of lung area involved in granulomatous lesions relative to total lung area used for morphometric analysis, n = 6 per group. Each point represents the mean of size of the lesions on each of six mice and its standard error.

Histological examination showed that several granulomas were visible in the BCG groups, mainly because they coalesced into what we termed “super lesions” (Fig. 3E–H). Compared with BCG + rAd-EGFP group (11.87% ± 9.5%), the BCG + rAd-hMASP-2 group displayed significantly enlarged pulmonary granulomas area in lung tissue (30.2% ± 17.4%, P = 0.023, Fig. 3I). The granulomatous pathologic feature showed more organized, stratified structure. ZN-stained lung recuts displayed massive presence of tuberculosis bacilli in the necrotised regions (Fig. 3J–L). The rich center of macrophage was surrounded by a number of lymphocytes which may be enclosed within a fibrous sheath that marked the periphery of the structure (Fig. 3).

### A higher degree of C4b deposition in hMASP-2 treatment group

**Figure 4:**
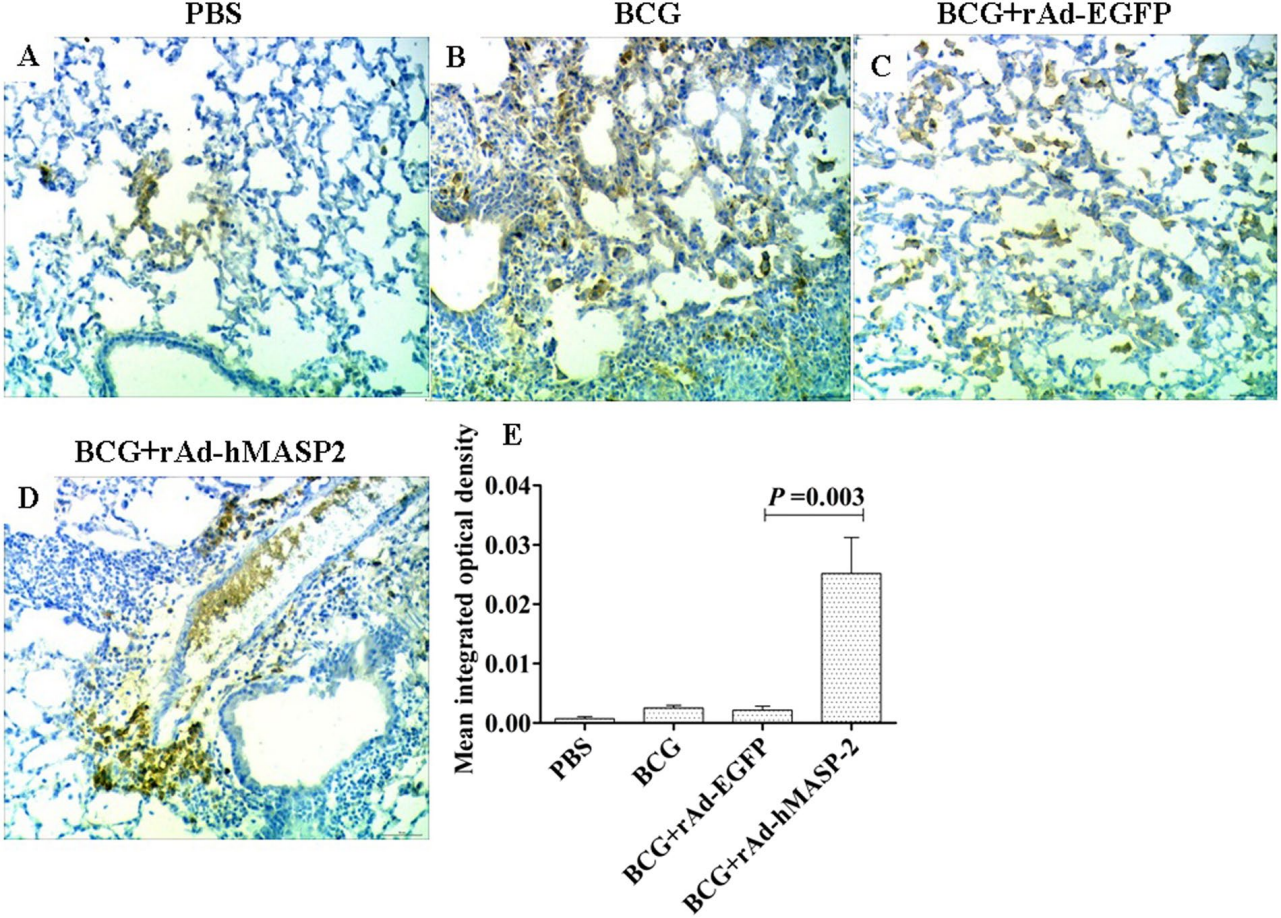
The lung tissue from BCG-infected BALB/c mice was stained for the deposition of C4 by immunohistochemistry for estimating the MASP-2 complex activity. (**A**~**D**) C4 deposition analysis of immunohistochemical stained lung sections from 4 representative mice (×200). As compared with other groups, the lung lesions tissue showing strong staining for C4 in rAd-hMASP-2 group. (**E**) Mean integrated optical density (IOD) of C4 deposition detemined by Image Pro-Plus according to the staining intensity by visual assessment, n = 4 per group. Each point represents the mean of four mice.

Deposition of C4b on the lung tissue was measured for the extent of hMASP-2 activation by immunohistochemistry. As shown in Fig. 4, BCG + rAd-hMASP-2 group showed a higher degree of C4b deposition compared with the BCG + rAd-EGFP group. Similarily, we assessed C4b deposition with immunohistochemical positive cell in different groups via relative quantitative way as detailed in Fig. 4. It was found that mean integrated optical density (IOD) of C4 deposition in BCG + rAd-hMASP-2 group was obviously higher than that of other groups in granulomas tissue (P < 0.05).

### hMASP-2 promoted macrophages migrating to the lungs

**Figure 5:**
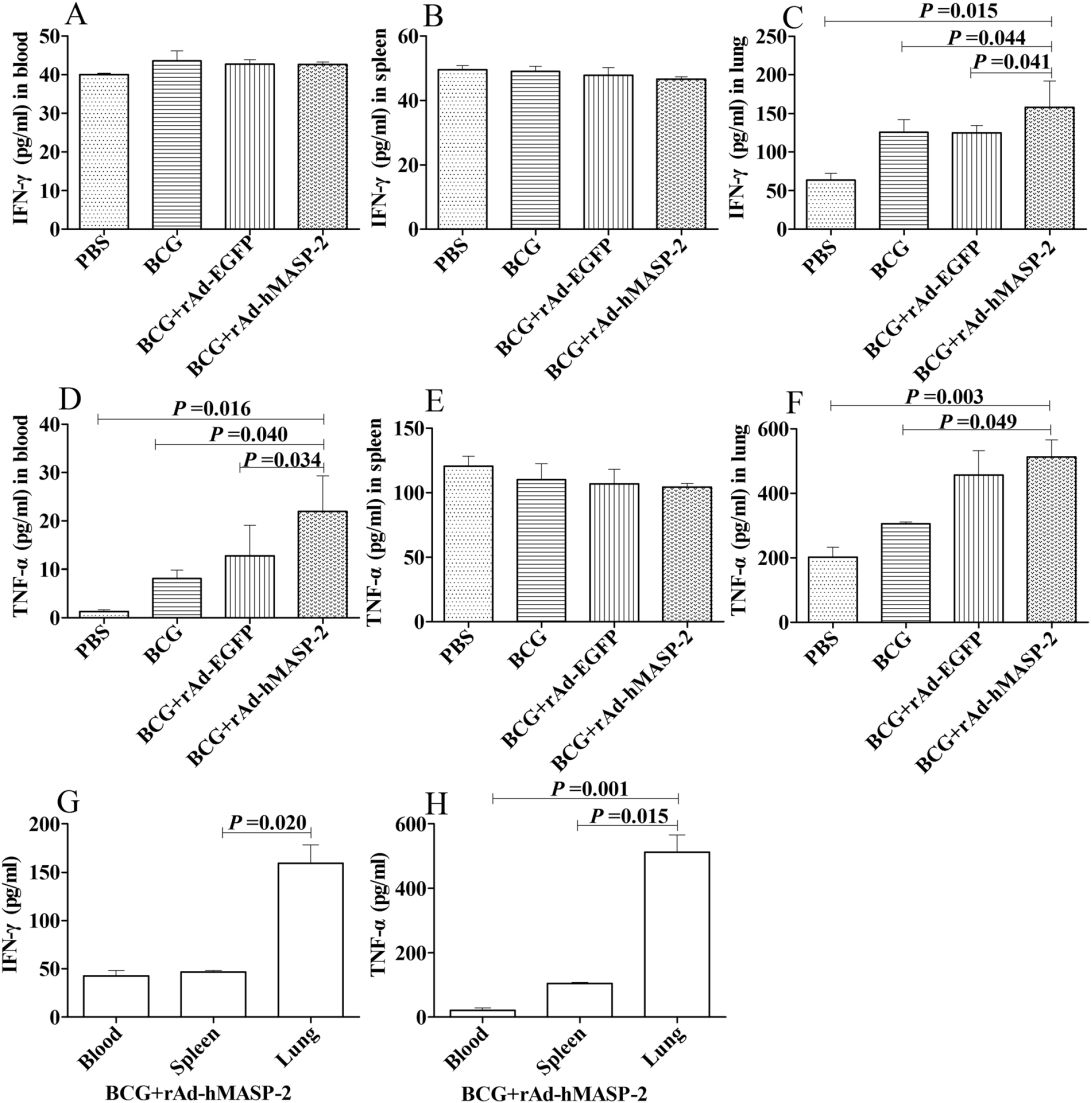
BALB/c Mice serum and tissue cytokine levels in each group. The levels of (**A**,**B**,**C** and **G**) IFN-γ and (**D**,**E**,**F** and **H**) TNF-α in the serum, lung and spleen were measured at 21 days in various groups, n = 6 per group. Each serum and tissue level represents the mean of 6 mice and its standard error.

At 21th day after the BCG infection, the role of hMASP-2 on macrophage accumulation in pulmonary granuloma was investigated with immunohistochemistry. A large increased number of CD11b^+^ macrophages were showed in BCG + rAd-hMASP-2 group. And compared with control groups, CD11b^+^ macrophages (optical density value: 0.0124 ± 0.0032) were obviously recruited in BCG + rAd-hMASP-2 group (P = 0.004, Fig. 5).

### hMASP-2 impacted on the mRNA expression for *il-6*, *ccl-12*, *il-12*, *il-8*, and cytokines of IFN-γ, TNF-α

**Figure 6:**
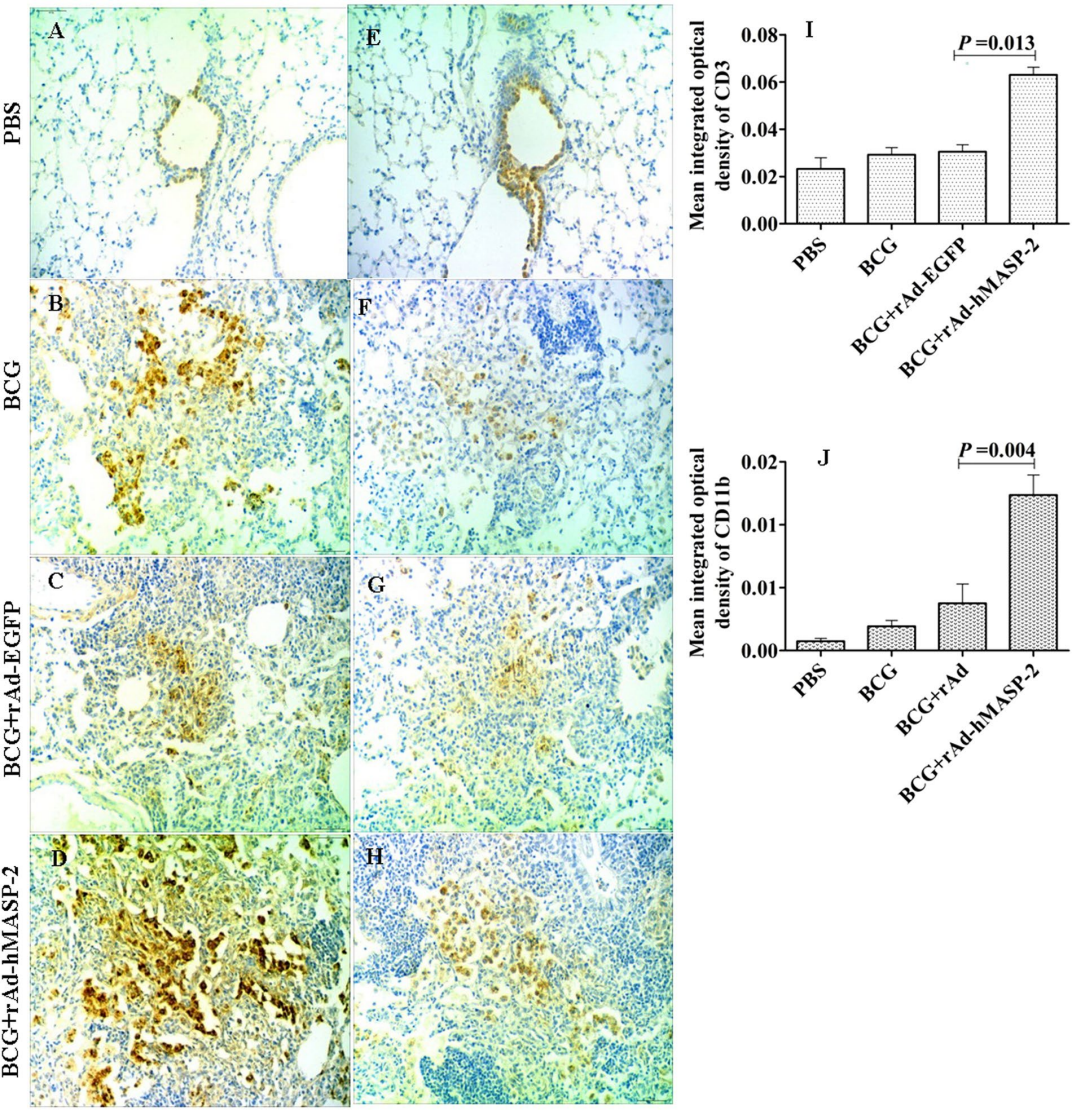
Lung tissue from BCG-infected BALB/c mice was stained for the presence of CD11b, CD3 by immunohistochemistry. (**A**~**D**) CD3 staining of infiltrating T cells in lung interstitium from 4 representative mice (×200). (**E**~**H**) CD11b staining of infiltrating macrophages from 4 representative mice. On day 21 after rAd-hMASP-2 administration, the lung lesions tissue showing strong staining for CD11b and CD3 in rAd-hMASP-2 group. (**I**,**J**) Mean integrated optical density (IOD) of CD11b, CD3 detemined by Image Pro-Plus according to the staining intensity by visual assessment, n = 5 per group. Data ± SD are from five independent experiments (n = 5).

The cytokines in mice showed obviously different between the local and systemic level. Thus, IFN-γ, TNF-α were evaluated in blood and lung as well as in spleen, and the mRNA expression for *il-6*, *ccl-12* were estimated in lung. In the rAd-hMASP-2 treated group, the levels of IFN-γ in lung and TNF-α in blood, were significantly increasing (P < 0.05, Fig. 6). The mRNA expression for *il-6*, *ccl-12*, and *il-12* was found to be much higher in the BCG + rAd-hMASP-2 group than that of the BCG + rAd-EGFP group (Fig. 6). In contrast, the mRNA levels for *il-8* were found to be much lower in the BCG + rAd-hMASP-2 group than that of the control group (Student’s t-test, P < 0.05, Fig. 6).

### hMASP-2 promoted lymphocytes migrating to the lungs

**Figure 7:**
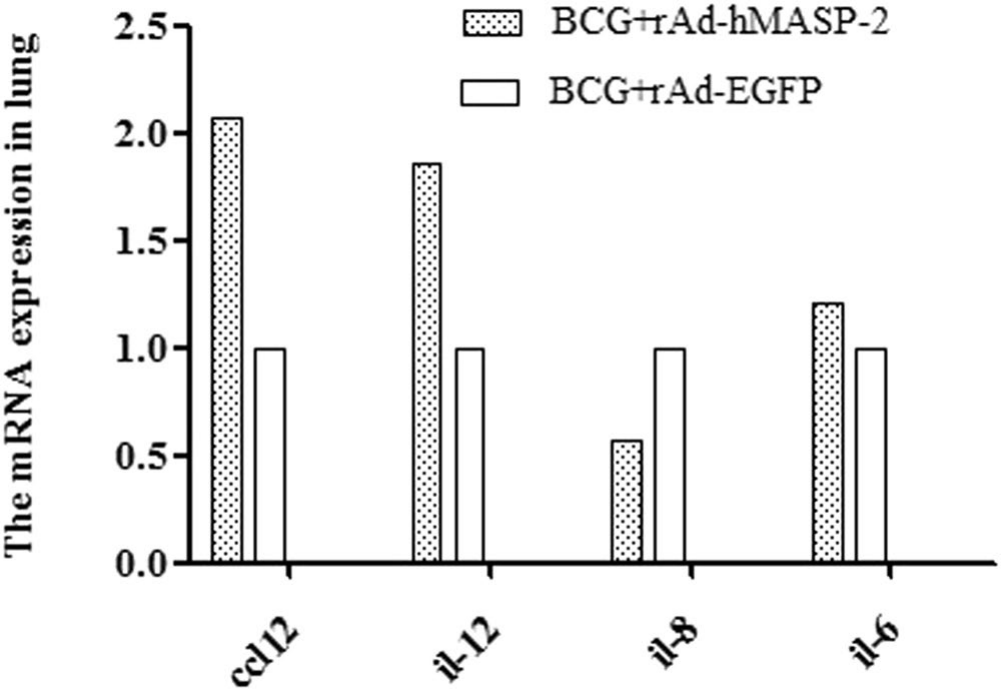
The mRNA expression for *il-6, ccl12, il12* and *il-8* in lung (n = 3 per group). Data represent mean values of conditions performed in duplicate.

**Figure 8:**
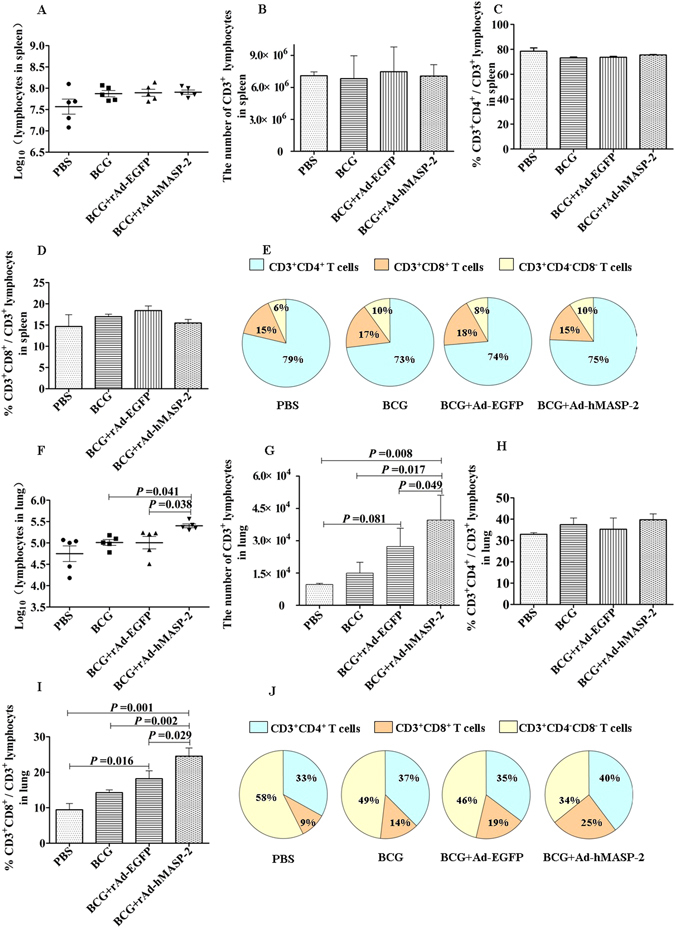
The number of lymphocytes and percentage of CD3^+^CD4^+^ and CD3^+^CD8^+^ cells within splenocytes (**A**~**E**) and pneumonocytes (**F**~**J**) in BCG-infecteded mice after treated intratracheally with rAd-hMASP-2. At day 21, isolated lung and spleen cells from mice were stained with PerCP-conjugated anti-CD3, PE-conjugated anti-CD4 andAPC-conjugated anti-CD8. CD3^+^CD4^+^ and CD3^+^CD8^+^ T-cell subgroup were detected via FCM, n = 5 per group. FACS analysis of T lymphocyte subsets showed no difference among various groups in total number of spleen lymphocytes (**A**) and CD3^+^ lymphocytes (**B**), percentage of CD3^+^CD4^+^ (**C**) and CD3^+^CD8^+^ (**D**) lymphocytes within splenocytes. (**E**) The CD3^+^ lymphocyte subsets distributed in the spleen of various groups. As compared with the rAd-EGFP group, (**E**) lung lymphocytes total number (2.56 × 10^5^ ± 6.41 × 10^4^) and (**F**) CD3^+^ lymphocytes (4.43 × 10^4^ ± 6.1 × 10^3^), (**H**) CD3^+^CD8^+^ lymphocytes percentage (24.55% ± 2.29%) in rAd-hMASP-2 group showed statistically significant increased. (**J**) The CD3^+^ lymphocyte subsets distributed in the lung of various groups. Data collected from five mice per group were expressed as means ± SD.

The distribution of CD3^+^T cells in pulmonary granulomas was detected via immunohistochemistry. As shown in Fig. 7, a large number of CD3^+^T cells were infiltrated in lung interstitium in rAd-hMASP-2 treated mice. The optical density value of CD3^+^T cells in the BCG + rAd-hMASP-2 group was 0.063 ± 0.0065, which was significantly higher than that of the control group (P = 0.013). A flow cytometric analysis of the CD3^+^T cells in lung showed that there was a significant increasing in the number of pulmonary infiltrating lymphocytes, including the population of CD3^+^CD8^+^ cell subsets in BCG-infected mice treated with rAd-hMASP-2, which was significantly higher than that in the others (P < 0.05, Fig. 8). Compared with the rAd-EGFP group, CD3^+^CD8^+^ lymphocytes percentage showed obviously increasing in rAd-hMASP-2 group, and CD3^+^CD4^−^CD8^−^ lymphocyte subsets declined markedly from 46% in rAd-EGFP group to 34% in rAd-hMASP-2 group (*P* = 0.011). Therefore, the CD3^+^CD8^+^T cells were the major effectors lymphocyte at this stage of the BCG infection in the lung. These cells are induced by rAd-hMASP-2 from an early stage of infection and maintained in the lungs against BCG.

## Discussion

The most significant phenotypic change that was observed in BCG-infected mice was the generation of protective granulomas, with *M. tuberculosis* localized in central cores. These granulomas were surrounded by immune cells, which ware triggered by the immune response in tuberculosis^28^. In this study, we found that the MASP-2 displayed an role in BCG-infected mice, which promoted both the progress of granuloma formaton and reduced the survival of the mycobacteria within lungs. Compared to the control group, MASP-2 treated group also showed increasement in survival rate. It suggested that MASP-2 showed a protective efficacy in targeting granuloma formation in the BCG-infected mice. Furthermore, there was a significant increasement in the number of pulmonary infiltrating immune cells, including the population of CD11b^+^ macrophages and CD3^+^ T cells, especially the CD3^+^ CD8^+^ T cell subsets in BCG-infected mice treated with MASP-2 group. The macrophages and lymphocytes which composed the structures of the granulomas are thought to be important effector cells encountered at the site of Mtb infection in the lung, and the prerequisite for the formation of granulomas^29^. Thus it indicated that MASP-2 may act as a immunomodulatory to promote macrophages and lymphocytes migrating to the lungs for the control of tuberculosis.

This increased number of immune cells in the lung not only inhibited bacterial growth but also regulated the formation and ongoing function of protective lesions^30^. It was reported that chemokines and cytokines induced by macrophages and lymphocytes was important in granuloma formation^31^. As the acute phase response factor, infected macrophages in the granuloma may be required for the surge of IL-6 in the mycobacteria-infected lung. Also, CCL group 12, a member of group CC chemokine family, is thought to attract monocytes and lymphocytes and coordinate cell movements during early immune response to pulmonary granulomas lesion^32^. Our data showed that expression of *ccl-12* at a higher level could be induced in rAd-hMASP-2mediated granuloma formation, which suggested that CCL-12 could attract monocytes and lymphocytes into granuloma and augment immune response to produce the protection response against BCG.

Moreover, IL-12 produced by macrophages was also high expression, which contributed to the host response to mycobacteria, improved the development of Th1 cells and the cytotoxic activity of CD8^+^ T lymphocytes^32^. The CD4^+^ Th1 cells ware activated and produced cytokines classically such as IL-2, IFN-γ and TNF-α, which involved in the protection role in tuberculosis^33^. Among Th1 type cytokines, IFN-γ and TNF-α were identified as the most important agents of the antimycobacterial cytokine cascade^34^. In current study, the levels of IFN-γ and TNF-α in lungs were significantly increasing in BCG-infected mice treated with rAd-hMASP-2. Therefore, IFN-γ and TNF-α played a critical role in the granuloma of *M. tuberculosis* infection through the activation of immune cells to produce effector molecules in lung. Interesting, the expression for *il-8* showed decreasing in BCG-infected mice treated with rAd-hMASP-2. IL-8, as inflammation factor, showed lower inflammatory symptoms.

In conclusion, the tuberculous granuloma was considered a host-beneficial role in the control of tuberculosis. MASP-2 displayed a potential protective role to promote the granuloma formation in lung and increase the survival of BCG-infected mice. The mechanism might be that the chemokines of CCL12 and Th1 type cytokines were induced in BCG-infected mice treated with rAd-hMASP-2, and then the T lymphocytes and macrophages were recruited to the granuloma site in control of tuberculosis.

## Materials and Methods

The study was approved by Experimental Animal Committee of Lanzhou University. The methods were carried out in accordance with the relevant guidelines and regulations of Experimental Animal Committee of Lanzhou University.

### rAd-hMASP-2 preparation

Human mannan-binding lectin associated serine protease-2 (hMASP-2) gene was amplified by PCR, digested with BamH II and EcoR II, and inserted into plasmid pYr-adshuttle-4(Invitrogen, USA). The constructed shuttle plasmid pYr-ads-4-hMASP-2 and pAd/PL-DEST adenovirus backbone vector was reconstructed through homologous recombination in mediation of LR enzyme. The obtained plasmid pAd-4-hMASP-2 was transfected to HEK293A cells for packing. The obtained recombinant adenovirus rAd-hMASP-2 was identified by restriction analysis, PCR and gene sequencing, and adjusted to the titer of 1.5 × 109 PFU/ml (plaque forming unit, PFU)^35^. BALB/c mice were infected with the recombinant adenovirus, and the expression of hMASP-2 mRNA in lung was determined by real-time fluorescent quantitative PCR. The recombined adenoviral vector of enhanced green fluorescent protein (rAd-EGFP) as negative control was prepared with the same method.

### *M. bovis* BCG

*M. bovis* BCG (BCG) Denmark strain (D2-PB302, a derivative of Copenhagen strain) was friendly gifted by Lanzhou University Institute of Tuberculosis. The strains was resuscitated at 37 °C in sauton potato medium. Then BCG was grown to log phase at 37 °C on Middlebrook 7H9 medium containing acid albumin-dextrose-catalase (ADC) supplement. And then were harvested by centrifugation, washed with ethanol and ethylether, adjusted to the concentration of 5 × 10^7^ CFU/ml (CFU are colony forming units of bacteria).

### Animals

BALB/c mice(license:SCXK(Gan)2010–0001)were purchased from the Lan Zhou Veterinary Research Institute, Chinese Academy of Agricultural Science and maintained in the SPF Grade Trial Animal Center (Lanzhou university, Lanzhou, China). All animals were female and 9–11 weeks old at the start of the experiments. Mice received free access to food and water throughout the study.

### Survival

All mice were infected one-time with 1 × 10^8^ CFU BCG by intranasal administration at day 0^36^ or with 50 μl of PBS as the control. 5 × 10^7^ PFU of rAd-hMASP-2, rAd-EGFP were respectively given to BCG + rAd-hMASP-2 group and BCG + rAd-EGFP group by intranasal at -3th, 4th and 11th days (Figure S1A).

### Bacterial infection and rAd-hMASP-2 treatment

The animals were received intranasal with 5 × 10^7^ CFU of BCG^37^ or 50 μl of PBS at day 0. Afterward, the mice were treated intranasal separately with 50 μl of rAd-hMASP-2 as MASP-2 group, and rAd (5 × 10^7^ CFU) as a control group at day -3th, 4th and 11th (Figure S1B). After treatment, those mice were sacrificed at 21th day, and the lungs, spleens, and serum were collected for experiments.

### Bacillary Loads

After the lungs were removed from mice, the number of viable bacterial count (expressed as colony forming units, CFU) in right lobes of lung were determined by spotting serial 10-fold dilutions of homogenates on Middlebrook 7H10 agar plates (containing 10% ADC) and ampicillin (0.01 mg/ml) for three weeks to calculate CFU^25^. Ziehl-Neelsen acid-fast staining was utilized to confirm that the bacterial colonies were tubercle bacilli.

### Histopathology analysis of lungs

The left lobes of lung were harvested from mice, fixed in 10% formalin, dehydrated in alcohol, embedded in paraffin blocks, sectioned at 5 μm thickness, the sections were then stained with haematoxylin-eosin (HE). Granulomas areas were quantitatively analyzed under low magnification with Image-Pro plus 6.0 software^38^. Histopathological changes on section were independently evaluated by three pathologists.

### Enzyme Linked Immunosorbent Assay (ELISA)

IFN-gamma (IFN-γ), TNF-alpha (TNF-α) in lung homogenates and serum were measured by ELISA (Dakewe, Hubei, China). The assay was performed and analysed following the manufacturer’s instructions. Briefly, Aliquots of 100 μL of serially diluted sera or lung homogenates were added respectively to wells of the ELISA plates, followed by further incubation for 1.5 h at 37 °C. After five washes with PBS containing 0.05% Tween 20 (PBS-T), the plates were incubated with horseradish peroxidase (HRP)-labeled goat anti-human immunoglobulin G (IgG) or IgM antibodies for 30 min at 37 °C. 3,3′,5,5′-tetramethylbenzidine (Sigma, St. Louis, MO, USA) was added at 100 μL/well after five washes with PBS-T, and the wells were incubated for 20 min at room temperature. Then, 50 μL of 2 M H_2_SO_4_ was added to each well to terminate the reaction, and the optical density (OD) was immediately read at 450 nm. The same normal control sera mixed in equal volumes served as negative controls^39^.

### Flow cytometry analysis of T-cell subpopulation in the lungs

Single pulmonary infiltrating (PIF) cells suspensions were prepared according to a previously established method^28^. Lymphocytes in right lobes of lung were separated from the PIF cells using 40% Percoll (Pharmacia, America). Single-cell suspensions of spleen were suspended in EZ-Sep TM mouse 1× Lymphocyte Separation Medium (Solarbio, China). The lymphocytes of suspensions were washed in PBS buffer and stained with PerCP-conjugated anti-CD3, PE-conjugated anti-CD4 and APC-conjugated anti-CD8(eBioscience, America). Flow cytometry was performed using BD FACSCalibur and the results were analysis with FlowJo 7.6.1.

### Immunohistochemistry

Paraffin-embedded tissue sections were rehydrated and stained with Rabbit Anti-mouse CD3 and CD11b (Abcam, England) and Biotin-Labeled Goat Anti-Rabbit IgG antibody (Abcam, England). Cryosections were stained with rat anti-mouse C4b mAb (Abcam, England) and Peroxidase-Conjugated Goat Anti-Rat IgGAb (ZSGB-BIO, USA). The stained sections were prepared for microscopic observation and histometry analysis using the Image-Pro Plus 6.0^38^.

### Fluorescence-based quantitative real-time PCR (RT-qPCR)

RT-qPCR was made to detect the mRNA expression of *ccl-12*, *il-12*, *il-8*, and *il-6* gene. The cDNA was reverse-transcribed from total RNA with 5× Reaction Mix and Script Reverse transcriptase (Bio-Rad, USA). The synthesized first-strand cDNA was amplified using Power SYBR Green PCR Master Mix (ABI, America). The amplified PCR products were quantified by YBR Green incorporation. The primers sequences of *ccl-12*, *il-12*, *il-8*, and *il-6* were showed in Table S1. All samples were analyzed in duplicate. Real-time PCR reactions were normalized to the Ct values for litchi LcActin (HQ615689)^40^. The relative expression levels of the target genes were calculated using the formula 2^−ΔΔCt^.

### Graphics and statistics analysis

All data are expressed as the mean ± standard deviation (SD), and the levels of significance are cited. SPSS statistical package version 19.0 for windows was used for all data analyses. The ANOVA analyses were combined with a least significant difference (LSD) test when appropriated. An independent-sample t-test was performed to assess significant differences between the treated and the respective control groups for the number of pups/dam analysis.

## Electronic supplementary material

Supplementary Information
