# Supplementary material for: Effects of hMASP-2 on the formation of BCG infection-induced granuloma in the lungs of BALB/c mice
Source: Sci Rep. 2017 May 23;7:2300. doi: 10.1038/s41598-017-02374-z (PMC5442121; doi:10.1038/s41598-017-02374-z)
Supplement: Supplementary file 1 — Supplementary Information [file 41598_2017_2374_MOESM1_ESM.doc]

Effects of hMASP-2 on the formation of BCG infection-induced granuloma in the lungs of BALB/c mice

Xiaoying Xu^1,4,+^, Xiaoling Lu^1,+^, Xingfang Dong^1^, Yanping Luo^1^, Qian Wang^1^ , Xun Liu^2^, Jie Fu^1^, Yuan Zhang^1^, Bingdong Zhu^2^, Xingming Ma^1,3^,*

Table S1 The primers sequences of *ccl2, il12, il8, il6 and β-actin*

| Gene | Forward primer (5'to3')  Reverse primer (5'to3') | Amplification  size | GenBank  Acc.No. |
| --- | --- | --- | --- |
| *ccl2*  (mice) | GGCTCAGCCAGATGCAGTTAA | 76bp | NM_011333.3 |
|  | CCTACTCATTGGGATCATCTTGCT |  |  |
| *il6*  (mice) | TCCTTCCTACCCCAATTTCC | 75bp | NM_031168.1 |
|  | TCTTGGTCCTTAGCCACTCC |  |  |
| *Il8*  (mice) | CCATGGGTGAAGGCTACTGT | 79bp | NM_011339.2 |
|  | CCGGTGGAAATTCCTTTTGT |  |  |
| *Il12*  (mice) | GGAAGAGTCCCCCAAAAGCT | 78bp | NM_001303244.1 |
|  | CAGCAAAGGTGTCATGATGAACTT |  |  |
| *β-actin*  (mice) | TGGCTCCTAGCACCATGAAGA | 71bp | NM_007393.3 |
|  | GCCACCGATCCACACAGAGT |  |  |

**
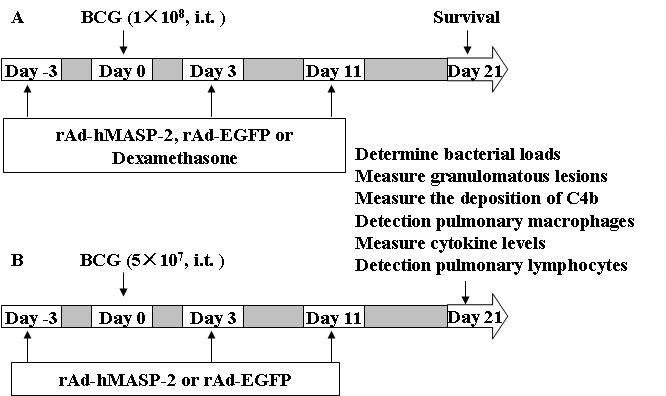
**

Figure S1 Experimental procedures. (A) The schedule of rAd-hMASP-2 treatment for survival. As the BALB/c mice were infected by intratracheally (i.t.) injection with 1×10^8^ CFU BCG, the mice were treated intratracheally with rAd-hMASP-2, rAd-EGFP or dexamethasone thrice at day -3, 0 and 11. rAd-EGFP and dexamethasone were used as control. The survival was observed within day 21 after infection of BCG. (B) The schedule of rAd-hMASP-2 treatment for detection. As the BALB/c mice were infected by intratracheally (i.t.) injection with 5×10^7^ CFU BCG, the mice were treated intratracheally with rAd-hMASP-2 or rAd-EGFP thrice at day -3, 0 and 11. rAd-EGFP was used as a control for rAd-hMASP-2.
